# Antioxidant Bibenzyl Derivatives from *Notholaena nivea* Desv.

**DOI:** 10.3390/molecules16032527

**Published:** 2011-03-17

**Authors:** Giuseppina Cioffi, Paola Montoro, Olga Lock De Ugaz, Antonio Vassallo, Lorella Severino, Cosimo Pizza, Nunziatina De Tommasi

**Affiliations:** 1 Dipartimento di Scienze Farmaceutiche e Biomediche, Università degli Studi di Salerno, Via Ponte Don Melillo,1 84084 Fisciano (SA), Italy; E-Mails: g.cioffi@unisa.it (G.C.); pmontoro@unisa.it (P.M.); pizza@unisa.it (C.P.); 2 Pontificia Universidad Catolica del Perù, Apartado, 1761, Lima, Peru; E-Mail: olock@pucp.edu.pe; 3 Dipartimento di Chimica, Università degli Studi della Basilicata, Viale dell’Ateneo Lucano,10 85100 Potenza, Italy; E-Mail: antonio.vassallo@unibas.it; 4 Dipartimento di Patologia e Sanità Animale, Sez. Tossicologia, Università di Napoli Federico II, Via Del Pino,1 80137, Napoli, Italy; E-Mail: lorella.severino@unina.it

**Keywords:** *Notholaena nivea*, bibenzyl derivatives, NMR, antioxidants activity

## Abstract

Four new bibenzyl derivatives were isolated, together with other known bibenzyls, by bioassay-guided fractionation of a CHCl_3_-MeOH extract of *Notholaena nivea* Desv. (Pteridaceae) aerial parts. The structures were elucidated by NMR, ESIMS and other spectral analyses. Their antioxidative effects towards superoxide, lipidic peroxidation and the 2,2’-azino-bis-3-ethilbenzothiazoline-6-sulfonic acid (ABTS) radical were assayed. Results showed that the compound 3,12-dihydroxy-5-methoxybibenzyl (**6**) is the most active compound in the ABTS free-radical scavenging test, while in the coupled oxidation of β-carotene and linoleic acid assay the compound 5,12-dihydroxy-3-methoxydibenzyl-6-carboxylic acid (**1**) exerted the highest activity after 1h. A superoxide anion enzymatic test was also carried out and the results were confirmed by an inhibition of xanthine oxidase activity assay. The putative protective role played by compounds **1** and **6** on the injurious effects of reactive oxygen metabolites on the intestinal epithelium, using a Caco-2 human cell line, was investigated. H_2_O_2_-induced alterations were prevented by preincubating the cells with compounds **1** and **6**.

## 1. Introduction 

Developments in biomedical science have shown that free radicals are involved in many diseases. They interact chemically with the unsaturated fatty acids in the biomembrane resulting in membrane lipid peroxidation, which is strongly connected to aging, carcinogenesis and atherosclerosis. Free radicals also attack DNA and cause mutations leading to cancer. In addition lipid peroxidation is an important deterioration reaction in the processing and storage of food. 

*Notholaena nivea* (Pteridaceae) is a South American plant, commonly known as Cuti Cuti. Its leaves can be used for the preparation of infusions or herbal teas. These teas are consumed in particular for the hypoglycaemic effect attributed to the green parts of the plant [1].

**Figure 1:**
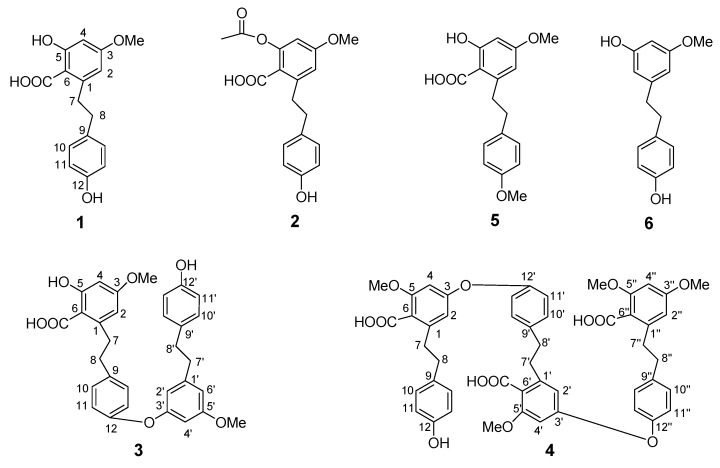
Compounds **1**-**6** from *Notholaena nivea*.

During our continuing search for plant-derived bioactive compounds, a CHCl_3_-MeOH extract of *Notholaena nivea* leaves was found to exhibit significant antioxidant effects in a bioautographic TLC assay [2]. Bioassay-guided fractionation of this extract utilizing antioxidation assays resulted in the isolation of four new bibenzyl and bisbibenzyl derivatives **1-4**, which were purified along with the known compounds 5-hydroxy-3,12-dimethoxy-6-carboxybibenzyl (**5**) and 3,12-dihydroxy-5-methoxybibenzyl (**6**) [3,4] (Figure 1).

The antioxidant activity recorded in this preliminary test were confirmed by the TEAC assay, in order to have a value indicating the free radical activity of each pure compound; moreover the activity of compounds **1-6** against the inhibition of lipid peroxidation according to autoxidation of linoleic acid and in the interaction with the xanthine-oxidase enzymatic system and superoxide anion product was assayed. The possible protective role played by compound **1** and **6** on the injurious effects of reactive oxygen metabolites on the intestinal epithelium, using a Caco-2 human cell line, whereby the H_2_O_2_-induced alterations were prevented by preincubating Caco-2 cells with compounds **1** (500 µmol/L) and **6** (250 µmol/L), was also investigated.

This paper thus deals with the structure elucidation of new compounds **1**-**4**, as well as the antioxidant evaluation of all isolated compounds measured by their radical scavenging activity in the radical cation 2,2’-azino-bis-(3-ethilbenzothiazoline-6-sulfonate) (ABTS) test [5,6], the coupled oxidation of β-carotene and linoleic acid assay [7,8], superoxide anion enzymatic generation assay [9,10] and xanthine oxidase (XOD) activity assay [9], and cytotoxicity assay in Caco-2 cells [11].

## 2. Results and Discussion

### 2.1. Structure elucidation of compounds ***1***-***6***

The ESIMS spectrum of **1** showed a peak at *m/z* 311 corresponding to the [M+Na]^+^ ion adduct and indicating the molecular formula C_16_H_16_O_5_. ^13^C-NMR DEPT confirmed the presence of 16 carbon atoms (Table 1 and Table 2). The spectral data of compound **1** showed a close similarity to those of notholaenic acid [3]. The NMR spectrum of compound **1** indicates that the molecule consists of one *p*-disubstituted benzene ring (δ_H_ 6.82 d, 2H, *J* = 8.5 Hz; δ_H_ 7.11 d, 2H, *J* = 8.5 Hz) and one tetrasubstituted benzene ring (δ_H_ 6.32, 6.26 br s). In addition, the ^1^H-NMR signals indicated the presence of one -OMe group (δ_H_ 3.80) and a -CH_2_-CH_2_- bond (δ_H_ 3.16 and 2.79) (Table 1). ESIMS/MS data further pointed to a carboxyl group, as a mass fragment *m/z* 267 [(M+Na)-44]^+^ was observed. The chemical shift of the -CH_2_ groups suggest their position as a linkage between the two benzene rings, thus leading to a dihydrostilbene skeleton, which is in accordance with the UV spectrum and literature data for bibenzyls [3,12]. On the other hand, the base peak in the ESIMS, appearing at *m/z* 107, can be explained by the fragment -CH_2_-C_6_H_4_OH; and a peak at *m/z* 181 shows the second stable portion of the original molecule. Thus, the second benzene ring thus bears the carboxyl group, one methoxyl group, as a well as an -OH group. The chemical shift of the two protons of this ring excludes their proximity to the carboxylic acid function. The relative position of substituents on the second benzene ring could be assigned by the observation of correlations in the HMBC and 1D-ROESY spectra [12]. HMBC correlations were observed between -OMe and C-3, C-2 and C-4, between H-7 and C-2, C-6, C-9. The position of the -OH and -OMe groups could be assigned by 1D-ROESY spectra, correlation peaks were detected between the signals of H-7 and H-2 and between H-2 and -OMe. From the foregoing evidence, the structure of compound **1** was established as 5,12-dihydroxy-3-methoxy-dibenzyl-6-carboxylic acid.

Compound **2**, C_18_H_18_O_6_, showing in ESIMS spectrum an ion at *m/z* 353 [M+Na]^+^, had to be an acetyl derivative of compound **1** (δ_H_ 1.95, s, 3H; δ_C_ 172.0) according to ^1^H- and ^13^C-NMR spectra. The analysis of the ^1^H, ^1^H-COSY, HSQC and HMBC spectra allowed the assignment of all ^1^H- and ^13^C-NMR signals (Table 1 and Table 2) [13,14]. Thus, structure of compound **2** was established as 5-acetyloxy-12-hydroxy-3-methoxybibenzyl-6-carboxylic acid.

A molecular formula of C_31_H_30_O_7_ was determined for bisbibenzyl **3** by ESIMS, showing a pseudomolecular ion peak at *m/z* 537 [M+Na]^+^ and from the ^13^C- and ^13^C-DEPT NMR spectra which afforded a total 31 resonances corresponding to four sp^3^ methylenes, 13 sp^2^ methines and 11 quaternary carbons including six carbons bearing oxygen atoms and one carboxylic group.

In the ^1^H-NMR spectrum of **3**, two 1,4-disubstituted benzene rings (a set of ring A signals: δ_H_ 6.83 and 7.14, and ring C signals: δ_H_ 6.81 and 7.09), one 1,3,5 trisubstituted ring B [δ_H_ 6.26, 6.24 and 6.18, br s] and one 1,3,5,6 tetrasubstituted ring D (δ_H_ 6.16 and 6.28) whose presence was reinforced by 1D TOCSY and COSY, HSQC, HMBC experiments were apparent (Table 1 and Table 2) [15,16]. The ^1^H-NMR spectrum of **3** also displayed the signals of four benzyl methylenes at δ_H_ 3.18, 3.20, 2.86, 2.82 corresponding to the ^13^C NMR signals at δ_C_ 38.0, 39.0, 38.6, 38.1 which are characteristic for bisbibenzyl derivatives. The arrangement of the substituents on the four benzene rings was established by HMBC experiments. Correlation peaks were observed between H-11’ and C-12’, C-9’ between H-11 and C-3’, C-12, C-9 between H-2 and C-7, C-6, C-3 between H-8 and C-10, C-1. From the foregoing evidence, the structure of compound **3** was established as 12-*O*-[3’-(5’-methoxy-12’-hydroxy)-bibenzyl]-5-hydroxy-3-methoxybibenzyl-6-carboxylic acid.

The molecular formula C_49_H_46_ O_13_ was assigned to compound **4** as shown by its ESIMS data ([M+ Na]^+^ *m/z* 865) in combination with the ^13^C-NMR spectral data. Compound **4** showed protons and carbons for six benzene rings, three of them 1,4-disubstituited and three of them 1,3,5,6-tetrasubstituted [17,18]. The ^1^H- and ^13^C-NMR data of **4** showed the signals of a trisbibenzyl derivative, and results from the oxidative combination of three bisbenzyl units. Assignment of the ^1^H- and ^13^C-NMR signals for each unit could be easy achieved by comparison with data of the compound **1** (Table 1 and Table 2) and by 1D TOCSY, HSQC and HMBC data.

From this evidence compound **4** had to be a trimeric derivative of compound **1**. The bonds between the three units were obtained from long range coupling of H-4 to C-3, C-6, C-2 respectively, between H-11’ and C-9’, C-10’, C-3 between H-2’ and C-4’, C-7’, C-6’ between H-11’’ and C-3’, C-9’’, C-12’’ (Table 1 and Table 2). Thus the structure of **4** was elucidated as 3-*O*-{12’-[12’’-*O*-(3’’,5’’-dimethoxy-6’’-carboxybibenzyl)]-5’-methoxy-6’-carboxybibenzyl}-12-hydroxy-5-methoxybibenzyl-6-carboxylic acid. The two known bibenzyl derivatives were identified as notholaenic acid (**5)** [3], and 3,12-dihydroxy-5-methoxybibenzyl (**6**) [19,20], by detailed MS and NMR analyses and comparison with literature data.

### 2.2. Free radical scavenging assay

Trolox equivalent antioxidant capacity (TEAC) has been used to determine the radical scavenging abilities of bibenzyl compounds **1-6**, as electron or H. donating agents throughout their ability to scavenge ABTS^+.^. The TEAC value resulting for compounds **1-6** are summarized in Table 3. Compounds **1-6** showed a good activity as radical scavengers.

### 2.3. Linoleic acid autoxidation assay

The antioxidative effect of compounds **1**-**6** from *Notholaena nivea* was also measured. The AA (antioxidant activity) values measured at t = 60 and t = 120 min for compounds **1**-**6**, employing bleaching of β-carotene as a model system are listed in Table 4. The data show that compounds **3**-**4** have no activity in this model, while compounds **1**-**2** and **5**-**6** showed a moderate activity, smaller than that of the synthetic antioxidant compound used as standard in this model.

### 2.4. Superoxide anion enzymatic generation assay

Superoxide anion is one of the most aggressive Reactive Oxigen Species (ROS) biosynthesized in the human organism. Superoxide anion destroys endothelium derived relaxing factor (EDRF) while its products, hydroxyl radicals and lipid peroxides, inhibit prostacyclin generation. Phenolic compounds like flavonoids have been shown to scavenge free radicals and their vasoprotective action has been associated with this particular property. Using an enzymatic biological generator of superoxide anion we have compared the free radical scavenging activity of compounds **1-6** with data reported in literature for other antioxidant compounds. The xanthine oxidase system generated superoxide anions as measured by the reduction of NBT (nitroblue tetrazolium ion), this reaction was inhibited from SOD (superoxide dismutase) in a concentration-dependent mode. All six investigated compounds inhibited the development of colour produced during the reaction of superoxide anion with NBT, with a moderate range of activity (Table 5).

### 2.5. Xanthine oxidase activity assay

With the aim of excluding the hypothesis that the superoxide anion scavenging activity was a result of an inhibition of xanthine oxidase enzymatic system, we have investigated the activity of the six compounds as inhibitors against the product of uric acid from xanthine in the oxidation reaction catalyzed from xanthine oxidase. We used a simple spectrophotometric assay, that allowed us to measure the production of uric acid from xanthine. Compounds **1**, **3**, **4** and **6** had no activity as direct inhibitor of xanthine oxidase, while compounds **2** and **5** showed a moderate activity that could partially account for the reduced production of superoxide anion (Table 5).

### 2.6. Effect of compound **1** and **6** on reactive oxygen metabolite-induced cytotoxicity

To investigate ROM-induced cytotoxic effects on differentiated Caco-2 cells, we added increasing amounts of H_2_O_2_ to the medium, bathing the apical side of the cells and after incubation we evaluated the cellular alterations after incubation. The overall cellular injury by means of the neutral red assay. Viable cells take up the dye by active transport, incorporating it into lysosomes, whereas nonviable cells do not; differences in the amount of neutral red incorporated by the cells indicated either a variation in the number of the cells. Incubation of cells in the presence of millimolar concentration of H_2_O_2_ resulted in a significant decrease in Caco-2 viability (Table 6) after 20 h of treatment with 10 mmol/L H_2_O_2_ we observed about 25% loss of cell viability. 

Then, this marker was used to verify the protective effects of compounds **1** and **6** against H_2_O_2_-induced injury to the intestinal Caco-2 cells. When cells were pretreated with compound **6** before being challenged with 10 mmol/L H_2_O_2_, no decrease in cell viability was observed, indicating that compound **6** at dose of 250 µmol/L suppresses the H_2_O_2_-induced toxicity at same dose compound **1** was inactive (Table 6).

## 3. Experimental 

### 3.1. General 

Optical rotations were measured on a Perkin-Elmer 241 polarimeter equipped with a sodium lamp (589 nm) and a 1 dm microcell. UV spectra were recorded on a Perkin-Elmer-Lambda 12 spectrophotometer. A Bruker DRX-600 NMR spectrometer, operating at 599.19 MHz for ^1^H and 150.86 MHz for ^13^C, using the UXNMR software package was used for NMR experiments; chemical shifts are expressed in δ (ppm) referring to the solvent peaks δ_H_ 3.34 and δ_C_ 49.0 for CD_3_OD and δ_H_ 2.49 and δ_C_ 39.5 for DMSO-*d_6_*; coupling constants, *J*, are in Hertz. DEPT ^13^C, 1D-TOCSY, 1D-ROESY, DQF-COSY, NOESY, HSQC, and HMBC NMR experiments were carried out using the conventional pulse sequences described in the literature. Column chromatography was performed over Sephadex LH-20 (Pharmacia, Uppsala, Sweden); HPLC separations were conducted on a Waters 600E chromatograph (Waters, Milford, MA, USA), equipped with a Waters 996 Photodiode Array Detector and µ-Bondapak C_18_ column (30 cm × 7.8 mm). ESIMS (positive mode) were obtained from LCQ DECA IT instrument equipped with an electrospray ion source and a ion trap, (Termo Electron, San Jose, CA, USA). UV spectra were recorded on a Perkin Elmer Lambda 12 Spectrophotometer.

### 3.2. Plant materials

Leaves of *Notholaena nivea* Desv. were collected from the Cusco region, Peru, in 2004. The plant material was identified by Prof. Olga Lock de Ugaz, Universidad Católica, Lima, Peru, and a voucher specimen has been deposited in author’s laboratory, voucher number 110.

### 3.3. Chemicals

ABTS (2,2’-azinobis-3-ethilbenzothiazoline-6 sulfonic acid), Trolox (6-hydroxy-2,5,7,8-tetra-methylchroman-2-carboxylic acid), potassium persulfate, linoleic acid, Tween 20, butylhydroxytoluene (BHT), β-carotene, EDTA, bovine serum albumin (BSA), nitroblue tetrazolium (NBT), xanthine, xanthine oxidase (XOD), sodium carbonate, sodium phosphate monobasic and sodium phosphate dibasic, neutral red, L-glutamine, hydrogen peroxide, quercetin were obtained from Sigma Aldrich (Gillingam, Dorset, U.K.). Dulbecco’s modified Eagle’s medium (DMEM), Eagle’s minimum essential medium (EMEM) and fetal calf serum (FCS) were purchased from Hyclone (Logan, UT, USA); penicillin-streptomycin, from porcine pancreas, PBS tablets were purchased from ICN-Flow (Costa Mesa, CA, USA).The solvents were obtained from Carlo Erba Reagent. Nanopure water was prepared by a Milli-Q apparatus.

### 3.4. Extraction and isolation

The air-dried powdered aerial parts of *Notholaena nivea* (Pteridaceae) (400 g) were defatted with *n*-hexane and successively extracted exhaustively for 48 h by maceration with CHCl_3_, CHCl_3_-MeOH (9:1) and MeOH (3 × 2 L), to give 15.0 g, 13.0 g, 8.5 g, and 11.0 g of residue, respectively. Each extract was tested for antioxidant potency, the CHCl_3_ –MeOH (9:1) was the most active and exhibited an IC_50_ value of 19 µg/mL, and was therefore chromatographed on Sephadex LH-20, using MeOH as eluent, to obtain 70 fractions of 10 mL combined together into 11 groups. Groups 7 and 8 demonstrated antioxidant activity (IC_50_ 28 µg/mL and 25 µg/mL) and were submitted to silica gel chromatography eluting with CHCl_3_ and increasing amounts of MeOH to obtain pure compound **1** (10 mg) together with 14 fractions. Fraction 10 was finally purified by RP-HPLC on a C-18 µ-Bondapack column (30 cm × 7.8 mm, flow rate 2.0 mL min^−1^) with MeOH-H_2_O (25:75) to yield **2** (12 mg) and **5** (6 mg). Groups 6 and 9 obtained from Sephadex LH-20, showed antioxidant activity and were fractionated over RP-HPLC on a C-18 µ-Bondapack column (30 cm × 7.8 mm, flow rate 2.0 mL min^−1^) with MeOH-H_2_O (7:3) for 6, MeOH-H_2_O (8:2) for 9, to afford respectively **4** (8 mg) from group 8; compound **6** (11.5 mg) from group 6, and **3** (6.4 mg) from group 9.

*Compound* **1**: UV (MeOH) λ_max_ 230, 279 nm; ESIMS (positive ion mode) *m/z* 311 [M+Na]^+^, 267 [M+Na-44]^+^; Elemental analysis: C 66.40%, H 5.58%, O 27.63%, calcd. for C_16_H_16_O_5_, C 66.66%, H 5.59%, O 27.75%; for ^1^H- and ^13^C-NMR data see Table 1 and Table 2.

*Compound* **2**: UV (MeOH) λ_max_ 230, 279 nm; ESIMS (positive ion mode) *m/z* 353 [M+Na]^+^; Elemental analysis: C 65.35%, H 5.45%, O 29.3%, calcd. for C_18_H_18_O_6_, C 65.45 %, H 5.49%, O 29.06%; for ^1^H- and ^13^C-NMR data see Table 1 and Table 2.

*Compound* **3**: UV (MeOH) λ_max_ 226, 280 nm; ESIMS (positive ion mode) *m/z* 537 [M+Na]^+^; Elemental analysis: C 72.31%, H 5.83%, O 21.68%, calcd. for C_31_H_30_O_7_, C 72.36%, H 5.88%, O 21.77 %; for ^1^H and ^13^C NMR data see Table 1 and Table 2. 

*Compound* **4**: UV (MeOH) λ_max_ 229, 275 nm; ESIMS (positive ion mode) *m/z* 865 [M+Na]^+^; Elemental analysis: C 69,79%, H 5.47%, O 24.66%, calcd. for C_49_H_46_O_13_, C69,82%, H 5.50%, O 24.68%; for ^1^H- and ^13^C-NMR data see Table 1 and Table 2. 

*Compound* **5**: UV (MeOH) λ_max_ 229, 275 nm; ESIMS (positive ion mode) *m/z* 325 [M+Na]^+^.

*Compound* **6**: UV (MeOH) λ_max_ 229, 275 nm, ESIMS (positive ion mode) *m/z* 267 [M+Na]^+^.

### 3.5. DPPH radical scavenging activity

The potential antioxidant activity of the extracts, fractions and pure compounds was determined on the basis of the scavenging activity of the stable 1,1-diphenyl-2-picrylhydrazyl (DPPH) free radical. Aliquots (30 mL) of methanolic solution containing each pure compound were added to a 0.004% MeOH solution of DPPH (3 mL). Absorbance at 517 nm, against a blank of methanol without DPPH, was determined after 30 min and the percent inhibition activity was calculated. IC_50_ values denote the concentration of sample required to scavenge 50% DPPH free radical. All tests were run in triplicate and averaged. The bioautographic TLC assay was performed as described by Cuendet *et al.* [2].

### 3.6. Autoxidation of ß-carotene

Heat-induced oxidation of an aqueous emulsion system of β-carotene and linoleic acid was measured by the method described by Pratt (1992) [7]. A quantity of linoleic acid (20 mg) and Tween 20 (200 mg) were placed in a flask, and a solution of β-carotene (2 mg) in CHCl_3_ (10 mL) was added. After removal of CHCl_3_, oxygenated distilled water (50 mL) was added. Aliquots of each compound (200 µL), dissolved in ethanol to a 15 µg/mL solution, were added to each flask with shaking. Samples without test compounds were used as blanks and sample with 2,6 di-*tert*-butyl-4-methylphenol (BHT) was used as a control substance. Samples were subjected to the oxidation, by placing them in an oven at 50 °C for 3 hours. The absorbance was red at 470 nm at regular intervals to monitor the rate of bleaching of ß-carotene. The antioxidant activities was expressed as AA, calculated with the equation Inhibitory ratio (AA) = 100 [1−A_0_−A_t_]/ A_00_−A_0t_; where A_0_ was the absorbance at the beginning of the incubation, with compound; A_t_ was the absorbance at the time t, with compound; A_00_ was the absorbance at beginning of the incubation, without compound; A_0t_ the absorbance at the time t, without compound. Each experiment was performed in triplicate.

### 3.7. Free radical scavenging assay

The TEAC value is based on the ability of the antioxidant to scavenge the ABTS^+.^. ABTS^+.^ cation radical was produced by the reaction between ABTS (7 mM) in water and potassium persulfate (2.45 mM), kept in the dark at room temperature for 12 hours. ABTS^+.^ is a blue-green cromogen with a characteristic absorption at 734 nm. The ABTS^+.^ solution was diluted with PBS, pH 7.4, to an absorbance of 0.70 at 734 nm and equilibrated at 30 °C.

Samples were diluted with methanol to have 0.3 mM, 0.5 mM, 1 mM, 1.5 mM, 2 mM solutions. The reaction was enhanced by the addition of diluted ABTS (1 mL) to 10 µL of each solution of sample or Trolox (standard), or 10 mL of methanol (blank). The determination was repeated three times for each sample solution. The percentage inhibition of absorbance at 734 nm was calculated for each concentration in function of the blank’s absorbance. The percentage inhibition was plotted as a function of compound or standard concentration. The antioxidant activities of compounds **1-6** are expressed as TEAC, Trolox equivalent antioxidant activity. TEAC value is defined as the concentration of standard Trolox solution with equivalent percentage inhibition to a 1 mM concentration solution of the compound after investigation.

### 3.8. Superoxide anion enzymatic generation assay

Superoxide anion was generated in an enzymatic system by preparing a mixture of xanthine and xanthine oxidase. The reaction mixture included 0.1 mM EDTA, 50 µg/mL bovine serum albumine (BSA), 25 µM nitroblue tetrazolium, 0.1 mM xanthine and 3.3 × 10^−3^ units xanthine oxidase (XOD) in 40 mM sodium carbonate buffer (pH 10.2) in a final volume of 3 mL. After incubation at 25 °C with increasing concentrations of samples, the absorbance of formazan produced was determined at 560 nm. 

The inhibitory effect of samples on the generation of superoxide anion were estimated by the equation: Inhibitory ratio = (A_0_−A_1_) × 100/ A_0_; where A_0_ absorbance with no addition of sample and A_1_ absorbance with addition of sample. Inhibitory ratio for each sample was plotted as a function of the concentration, then was calculated the IC_50_ value, with the statistical method of linear regression.

### 3.9. Xanthine oxidase inhibition assay

Xanthine oxidase inhibition activity was evaluated by the spectrophotometric measurement of the formation of uric acid by xanthine. A 100 µM solution of xanthine in 0.1 M phosphate buffer pH 7.8 with 0.04 units/mL of xanthine oxidase was incubated for 10 min at room temperature and read at 295 nm against a blank sample. Various concentrations of testing compounds were added to samples before the enzyme has been instilled and their effect on the generation of uric acid was used to calculate regression lines and IC_50_ values.

### 3.10. Cell cultures

The Caco-2 cells strain was obtained from Prof. A. Leone research group (Dipartimento di Scienze Farmaceutiche, Sezione Biologica, Università degli Studi di Salerno, Fisciano, Italy) and used between passages 75 to 90. The cells were routinely maintained in DMEM, containing 200 mL/L FCS, 10 mL/L of 100× nonessential aminoacids, 2 mmol/L L-glutamine, 5 × 10^4^ IU/L penicillin, 50 mg/L streptomycin at 37 °C in a 5% CO_2_ atmosphere at 90-100% relative humidity. Cell were grown in 10-cm petri dishes. For experiments, cells were seeded at a density of 90,000 cells/cm^2^ in a Transwell insert, and the medium (0.1 mL in the insert and 0.8 mL in the well) was changed every 48 h. Fourteen to sixteen days after confluence, the integrity of the monolayer of differentiated cells was monitored according to the method of Hildago *et al.* [21].

### 3.11. Induction of oxidative stress

An iron-free medium (EMEM) was used for the oxidative stress induction experiments. The oxidative stress was induced in the apical compartment of the transwell insert by addition of H_2_O_2_. After 20 h of incubation, several oxidative stress markers were measured. To assay the capacity of these compounds to protect Caco-2 cells from ROM-mediated oxidative injury, cells were preincubated for 4 h with compounds, which had been added to the apical side of monolayer. After the end of preincubation time, the medium was changed before the addition of the of the oxidative stress-inducing agents.

### 3.12. Neutral red assay

We assessed the cytotoxicity of ROM on Caco-2 by the viability test of neutral red uptake, performed according to the procedure of Fautz *et al.* [22]. After oxidative stress induction the medium in the insert was removed and replaced with 0.1 mL of fresh medium containing 1.14 mmol/M neutral red. At the end of 3 h of incubation, the medium was removed and cells were washed twice with PBS; finally the incorporated neutral red was released from cells by incubation for 15 min at room temperature in the presence of 1 mL of cell lysis buffer containing acetic acid (1%, v/v) and ethanol (50% v/v). To measure the dye taken up, the cell lysis products were centrifuged and supernatants spectrophotometrically measured at 540 nm. 

## 4. Conclusions

A fascinating hypothesis raised in the past few years is that the health-promoting action of some foods could be due to the presence of nonessential components, such as polyphenols (many with antioxidant potential) that could contribute to the modulation of the oxidative balance *in vivo* [23]. On the other hand reactive oxygen species (ROS) are considered related to many diseases, including atherosclerosis, liver injury, aging, inflammation, neurovegetative diseases and cancer. In the search for new biological active metabolites from natural sources, special interest has been focused on herbal products reputed in traditional medicine to have beneficial effects in inflammatory and cancer diseases. In the context of our research on the pharmacological properties of South American medicinal plants, six bisbibenzyl and bibenzyl derivatives from *Notholaena nivea* have been studied. While a large number of references are reported in literature about antioxidant activity of other classes of polyphenols, such as flavonoids [23,24], few references are reported for this class, and most of them regarding resveratrol and its derivatives [25,26], and there are few reports for bibenzyls related to these natural products. The present study deals with the activity of these compounds in several *in vitro* systems to evaluate the mechanism of their antioxidative effects. We have used two different models for studying free radical scavenging activity of bibenzyl compounds, the first model (DPPH) was used only in order to have a rapid bio-assay fractionation.

The second model gave us the value of this activity for each isolated compound. In this model we have used two different control samples, one of them was dihydroresveratrol, chosen because of the structural similarity with our compounds, and the other was quercetin, a compound extensively studied for its radical scavenging activity. Compounds **1** and **6** are the most active compounds, showing good activity respect to the standard, the TEAC values are similar to those showed by dihydroresveratrol, and slightly less than quercetin. In the model of autoxidation of linoleic acid, compounds **1-6** showed appreciable activity at concentration of 15 µg/mL, in comparison with BHT, used as standard. Activity of dihydroresveratrol was also measured at the same concentration, showing a lower activity. In the superoxide anion generation assay, the bibenzyls isolated from *N. nivea* have a low activity, when compared with the activity of dihydroresveratrol, probably due to the presence on the structure of a carboxylic group. On the other hand it is important to observe that none of compounds **1-6** is active in direct inhibition of xanthine oxidase; the small effect seen, seem due to the scavenging activity of superoxide anion.

In the second part of our study we investigated the possible protective effect of compounds **1** and **6** against reactive oxygen species-induced Caco-2 cytotoxicity. The incubation of cells in the presence of mM concentration of H_2_O_2_ results in a significant reduction of cell viability as measured by the neutral red assay. Compounds **6** is the most active, showing the ability to prevent H_2_O_2_-induced cytotoxicity at 250 µmol/L. In the system investigated, compound **1** exerted a protective effect up to a concentration of 250 µmol/L indicating that the 3-hydroxy group free located at the *para* position respect to the carboxyl function is more effective for the antioxidant activity. The data reported in this paper represent the first direct evidence that bibenzyl derivatives at µM concentration plays a protective role against ROM-induced oxidative injury, using a cell culture model as the experimental system.

## Figures and Tables

**Table 1:** ^1^H NMR Data of Compounds **1**-**4** (CD_3_OD, 600 MHz in methanol-*d*_4_). *^a^*

	1	2	4		3
Position	δ_H_	δ_H_	δ_H_	Position	δ_H_
1	-	-	-	1	-
2	6.26 br s	6.28 br s	6.18 br s	2	6.16 br s
3	-	-	-	3	-
4	6.32 br s	6.33 br s	6.26 br s	4	6.28 br s
5	-	-	-	5	-
6	-	-	-	6	-
7	3.16 t *J* = 6.0	3.18 t *J* = 6.0	3.20 t *J* = 6.0	7	3.18 t *J* = 6.0
8	2.79 m	2.80 m	2.80 m	8	2.86 m
9	-	-	-	9	-
10,14	7.11 d *J* = 8.5	7.12 d *J* = 8.5	7.10 d *J* = 8.5	10,14	7.14 d *J* = 8.5
11,13	6.82 d *J* = 8.5	6.82 d *J* = 8.5	6.83 d *J* = 8.5	11,13	6.83 d *J* = 8.5
12	-	-	-	12	-
-OMe	3.80 s	3.81 s	3.74 s	-OMe	3.76 s
COOH	-	-	-	COOH	-
MeCO-		1.95 s	-	MeCO-	
MeCO-		-	-	MeCO-	
1’			-	1’	-
2’			6.16 br s	2’	6.18 br s
3’			-	3’	-
4’			6.28 br s	4’	6.26 br s
5’			-	5’	-
6’			-	6’	6.24 br s
7’			3.18 t *J* = 6.0	7’	3.20 t *J* = 6.0
8’			2.86 m	8’	2.82 m
9’			-	9’	-
10’, 14’			7.15 d *J* = 8.5	10’, 14’	7.09 d *J* = 8.5
11’, 13’			6.83 d *J* = 8.5	11’,13’	6.81 d *J* = 8.5
12’			-	12’	-
-OMe			3.76 s	-OMe	3.76 s
COOH			-		
1’’			-		
2’’			6.14 br s		
3’’			-		
4’’			6.23 br s		
5’’			-		
6’’			-		
7’’			3.18 t *J* = 6.0		
8’’			2.80 m		
9’’			-		
10’’, 13’’			7.08 d *J* = 8.5		
11’’, 14’’			6.85d *J* = 8.5		
12’’			-		
-OMe			3.77 s		
-OMe			3.76 s		
COOH			-		

*^a^ J* values are in parentheses and reported in Hz; chemical shifts are given in ppm; assignments were confirmed by DQF-COSY, 1D-TOCSY, HSQC, and HMBC experiments.

**Table 2:** ^13^C NMR Data of Compounds **1**-**4** (CD_3_OD, 600 MHz in methanol-*d*_4_). ^*a*^

	1	2	4		3
Position	δ_C_	δ_C_	δ_C_	Position	δ_C_
1	149.0	148.7	148.4	1	144.3
2	106.2	107.0	107.0	2	106.6
3	166.0	166.8	162.0	3	162.9
4	101.0	100.1	99.8	4	99.7
5	166.0	165.3	163.0	5	163.8
6	111.2	109.8	112.0	6	109.8
7	36.5	36.2	38.2	7	38.0
8	38.0	37.8	38.4	8	38.6
9	137.5	137.0	136.0	9	135.9
10,14	129.5	129.2	131.0	10,14	130.4
11,13	116.0	115.5	114.8	11,13	114.6
12	156.2	155.2	156.3	12	156.3
-OMe	57.4	57.1	57.1	-OMe	57.3
COOH	175.0	174.6	175.0	COOH	174.0
MeCO-		21.0	-	MeCO-	-
MeCO-		172.0	-	MeCO-	-
1’			147.9	1’	148.5
2’			109.7	2’	111.0
3’			161.5	3’	165.4
4’			100.0	4’	109.7
5’			163.0	5’	161.3
6’			110.8	6’	100.0
7’			38.0	7’	38.1
8’			39.0	8’	39.0
9’			136.9	9’	136.5
10’, 14’			130.0	10’, 14’	131.0
11’, 13’			115.0	11’,13’	114.0
12’			159.0	12’	157.8
-OMe			57.0	-OMe	56.9
COOH			174.5		
1’’			148.0		
2’’			109.9		
3’’			161.0		
4’’			99.8		
5’’			163.9		
6’’			111.0		
7’’			37.8		
8’’			38.6		
9’’			136.5		
10’’, 13’’			130.1		
11’’, 14’’			114.4		
12’’			158.0		
-OMe			57.3		
-OMe			57.5		
COOH			174.5		

*^a^ J* values are in parentheses and reported in Hz; chemical shifts are given in ppm; assignments were confirmed by DQF-COSY, 1D-TOCSY, HSQC, and HMBC experiments.

**Table 3:** TEAC values for compounds **1**-**6** of *N. nivea*. ^a^

Compounds	TEAC (μM)
**1**	1.98 ± 0.05
**2**	1.38 ± 0.01
**3**	1.22 ± 0.02
**4**	1.50 ± 0.03
**5**	1.21 ± 0.01
**6**	2.55 ± 0.02
Dihydroresveratrol	2.30 ± 0.07
Quercetin	2.91 ± 0.02

^a^ Each analysis was performed in triplicate. Values are means ± SD.

**Table 4:** Linoleic acid autoxidation inhibition of compounds **1**-**6** of *N. nivea*. ^a^

Compounds	1 h	2 h
BHT	60.00%	51.07%
**1**	32.60%	11.70%
**2**	28.51%	19.16%
**3**	0	0
**4**	0	0
**5**	26.56%	19.72%
**6**	29.35%	7.31%
Dihydroresveratrol	16.11%	20.12%

^a^ Values are means of three repetitions. Standard deviation for all analyses were ≤ 1.

**Table 5:** Superoxide anion scavenging activity and xanthine oxidase activity inhibition of compounds **1**-**6** of *N. nivea*. ^a^

Compounds	Superoxide anion scavenging activity IC_50_ (μM)	Xanthine oxidase activity inhibition IC_50_ (μM)
**1**	96.93 ± 0.42	>100
**2**	81.11 ± 0.68	71.39 ± 0.45
**3**	78.16 ± 1.15	>100
**4**	81.32 ± 2.01	>100
**5**	58.35 ± 1.18	63.98 ± 2.13
**6**	83.33 ± 1.06	>100
Dihydroresveratrol	60.88 ± 1.12	>100

^a^ Values are means of three repetitions ± SD.

**Table 6:** Effect of compounds **1** and **6** on H_2_O_2_-induced cytotoxicity in Caco-2 cells. ^a^

Compounds	Concentration	cell viability
Control	-	100 %
H_2_O_2_	+ 10 mmol/L	75%
**1**	+ 500 μmol/L	88%
	+ 250 μmol/L	80%
**6**	+ 250 μmol/L	98%
	+ 125 μmol/L	90%

^a^ All the variables were tested in three independent cultures for each experiment and each experiment was repeated three times (n = 9). Values are means ±SD. Level of significance: P < 0.05. Cells incubated only in presence of compounds **1** and **6** showed neutral red uptake values similar to those of untreated cells.
